# Expression of miR-33 from an *SREBF2* Intron Targets the *FTO* Gene in the Chicken

**DOI:** 10.1371/journal.pone.0091236

**Published:** 2014-03-13

**Authors:** Fang Shao, Xingguo Wang, Jianfeng Yu, Honglin Jiang, Bin Zhu, Zhiliang Gu

**Affiliations:** 1 Department of Life Science and Technology, Changshu Institute of Technology, Changshu, Jiangsu, China; 2 College of Basic Medicine and Biological Science, Soochow University, Suzhou, Jiangsu, China; 3 Department of Animal and Poultry Sciences, Virginia Polytechnic Institute and State University, Blacksburg, Virginia, United States of America; University of Massachusetts Medical, United States of America

## Abstract

The sterol regulatory element binding transcription factor 2 (*SREBF2*) gene encodes a transcription factor that activates the expression of many genes involved in the synthesis and uptake of cholesterol, fatty acids, triglycerides, and phospholipids. Through bioinformatics, we found that intron 16 of the chicken *SREBF2* gene might encode the chicken miR-33. Using quantitative RT-PCR, we detected the expression of miR-33 in a variety of chicken tissues including skeletal muscle, adipose tissue, and liver. Three hundred and seventy eight genes were predicted to be potential targets of miR-33 in chickens via miRNA target prediction programs “miRanda” and “TargetScan”. Among these targets, the gene *FTO* (fat mass and obesity associated) encodes a Fe(II)- and 2-oxoglutarate-dependent nucleic acid demethylase that regulates lipid metabolism, and the possibility that its expression is negatively regulated by miR-33 in the chicken liver was therefore further studied. Co-transfection and dual-luciferase reporter assays showed that the expression of luciferase reporter gene linked to the 3′-untranslated region (3′UTR) of the chicken *FTO* mRNA was down-regulated by overexpression of the chicken miR-33 in the C2C12 cells (*P*<0.05). Furthermore, this down-regulation was completely abolished when the predicted miR-33 target site in the *FTO* 3′UTR was mutated. In contrast, the expression of *FTO* mRNA in the primary chicken hepatocytes was up-regulated after transfection with the miR-33 inhibitor LNA-anti-miR-33. Using quantitative RT-PCR, we also found that the expression of miR-33 was increased in the chicken liver from day 0 to day 49 of age, whereas that of the *FTO* mRNA was decreased during the same age period. These data together suggest that miR-33 might play an important role in lipid metabolism in the chicken liver by negatively regulating the expression of the *FTO* gene.

## Introduction

In addition to classical transcription factors, a new class of non-coding RNAs termed microRNAs (miRNAs) has emerged as critical regulators of gene expression acting predominantly at the posttranscriptional level. miRNAs are single-stranded small RNA molecules, with the length of 18∼25 nucleotides (nt). They bind to the 3′-untranslated regions (3′UTR) of mRNA transcripts to reduce the translation of these transcripts or to cause their degradation [1]. Bioinformatics predictions and experimental approaches indicate that a single miRNA may target more than 100 mRNAs [2]. In a genome, 20%∼30% genes are regulated by miRNAs [3]. miRNAs have been implicated in the regulation of almost all developmental, physiological and pathological processes [4].

microRNA-33 (miR-33) is transcribed from an intronic region within the sterol response element binding transcription factor 2 (*SREBF2*), also called sterol response element binding protein-2 gene [5], which directly activates the expression of more than 30 genes involved in the synthesis and uptake of cholesterol, fatty acids, triglycerides, and phospholipids [6], [7]. miR-33 is expressed in numerous mammalian cell types and tissues [8], [9]. The expression levels of miR-33 and *SREBF2* are closely paralleled in human or mouse hepatocytes and macrophages [5], [10], suggesting that they are coregulated at the transcriptional level. Research by multiple groups has shown that miR-33 analogs regulate cholesterol and fatty acid metabolism in mammalian systems, corresponding with the function of its host gene [10], [11]. A number of miR-33 targets have been identified, including the *ABCA1*, *ABCG1* and *NPC-1* genes, which are involved in cholesterol efflux and high-density lipoprotein metabolism [5], [8], [11], and the *CPT1A*, *CROT* and *HADHB* genes, which are involved in fatty acid β-oxidation [11]. In addition to regulating cholesterol transport, high-density lipoprotein metabolism and fatty acid β-oxidation, miR-33 was recently reported to regulate cell cycle progression and cellular proliferation [12], inflammatory response [13] and insulin signaling [14].

Genome-wide association studies (GWAS) have initially identified the *FTO* gene as a gene strongly associated with obesity [15]. Bioinformatics analyses suggest the human FTO is a member of the non-heme dioxygenase (Fe(II)- and 2-oxoglutarate–dependent dioxygenase) superfamily [16], [17], that catalyze demethylation of 3-methylthymine and 3-methyluracil in single-stranded DNA and RNA, respectively [18]. Based on its crystal structure FTO has no appreciable activity on double stranded nucleic acids, and it has a substrate preference for methylated RNA over DNA [19]. More recently, Jia et al. reported that N6 methyl adenosine (6meA) in both DNA and RNA is another substrate of FTO [20]. The *FTO* gene is conserved in various vertebrate species including fish and chicken [21]. Using transgenic mouse models, in which the function of FTO is either enhanced [22] or eliminated [23], it was found that FTO plays an important role in food intake and energy metabolism.

The objectives of this study were to determine whether miR-33 is expressed in the chicken, and, if so, to identify its target genes. In this paper, we provide computational and experimental evidence demonstrating that miR-33 is expressed in the chicken. We also provide evidence suggesting that miR-33 may regulate the expression of the *FTO* gene in the chicken liver.

## Materials and Methods

### Computational Prediction of miR-33 Target Genes

The 3′UTR sequences of *gallus gallus* were downloaded from the 3′UTR database (http://utrdb.ba.itb.cnr.it/). The miRNA target prediction software miRanda, miRDB (http://mirdb.org/miRDB) and targetscan (http://www.targetscan.org/) were employed to predict miR-33 binding sites in chicken 3′UTRs.

### Construction of Plasmids

A DNA fragment containing the predicted miR-33 and 150 bp upstream and 150 bp downstream sequences was amplified by PCR from chicken genomic DNA. The PCR product was cloned into the pcDNA3.1 (+) vector (Invitrogen, Carlsbad, CA) at the HindIII and XhoI restriction sites to generate the chicken miR-33 over-expression vector pcDNA3.1-miR-33. A negative control vector pcDNA3.1-NC-miRNA was constructed by inserting into pcDNA3.1 a sequence that had no predicted target site in the chicken *FTO* 3′UTR. The chicken *FTO* 3′UTR encompassing the predicted miR-33 binding site was amplified by PCR and directionally inserted downstream of the luciferase expression cassette of the pMIR-reporter vector (Ambion, Carlsbad, CA) at the SacI and HindIII sites to construct the pMIR-FTO reporter vector. Point mutations in the seed region of the predicted miR-33 binding sequence within the 3′UTR of chicken *FTO* were generated using overlap-extension PCR, and the resulting plasmid was named pMIR-FTOmut. All constructs were confirmed by sequencing and prepared to reduce endotoxin by using the PureLink™ HiPure Plasmid Filter Purification Kits (Invitrogen, Carlsbad, CA, USA).

### RNA Isolation and Real-time qRT-PCR

Arbor Acres commercial chickens were used in the present study. Various tissues were collected from 4-week-old chickens and liver samples were taken from 0, 1, 2, 3, 4, 5, 6 and 7-week-old chickens following euthanasia. All procedures involving chickens were approved by the Changshu Institute of Technology Institutional Animal Use and Care Committee. Total RNAs were isolated using TRIzol reagent (Invitrogen, Carlsbad, CA) according to the manufacturers’ protocol, and RNA concentrations and integrity were determined by NanoDrop ND2000 spectrophotometry (Thermo Scientific, Wilmington, DE) and formaldehyde-agarose gel electrophoresis, respectively. The expression of miR-33 was quantified by real-time qRT-PCR according to the protocol of TaqMan MicroRNA Assay (Applied Biosystems, Foster City, CA). All reactions were performed in duplicate. The threshold cycle (Ct) was defined as the fractional cycle number at which the fluorescence passes the fixed threshold. Ct values for a miRNA were normalized to that for 18S rRNA. The expression of mRNA was quantified by real-time qRT-PCR using the PrimeScript RT kit, and SYBR Green PCR master mix (Takara, Dalian, China). The Ct values for an mRNA were normalized to those for *β-actin* mRNA. The sequences of primers for this study are listed in Table 1.

**Table 1:** Primer sequences for plasmid construction and real-time qRT-PCR.

Primername	Primer sequences (5′–3′)	Productslength/bp	Tm(°C)	Purpose
ggamiR33	F/R cccaagcttCTCCATTTCAGGCAGCATCG/ccgctcgagCCAAATCCCTTTTCCCCATC	350	58	Cloning
ggaFTO	F/R cgagctcTCAGTAGGTAGGATATCAGG/cccaagcttATCCATGGGCTACAAGGTCA	288	58	Cloning
ggaFTOm	F/R GTGCTTCATTCGAAATTCTATTGGTTTCCACC/GGTGGAAACCAATAGAATTTCGAATGAAGCAC	288	58	Cloning
ggaFTO	F/R TAGTGATTGGAACCTGAAGG/CATCAAGCATCAAGTAGAGG	128	58	qRT-PCR
ggasrebp2	F/R AGCCTCAGATCATCAAGACG/TTCCATTGCTCCCAACAAGG	153	58	qRT-PCR
β-actin	F/R CACGGTATTGTCACCAACTG/ACAGCCTGGATGGCTACATA	200	58	qRT-PCR

### Isolation and Culture of Primary Chicken Hepatocytes

Hepatocytes were isolated from four-week-old chickens using an improved two-step collagenase method as described before [24]. In brief, chickens were fasted 12 h before being anaesthetized by intraperitoneal injection of natrium thiopenthal (50 mg/kg BW) and anticoagulated by intraperitoneal injection of heparin (1,750 U/kg BW). Livers were first perfused with 250 ml of buffer A (5 mM EDTA, 10 mM of HEPES, 137 mM of NaC1, 3 mM of KCl, 3 mmol/L of Na_2_HPO_4_, pH 7.5) and then with 250 ml of buffer B (buffer A without EDTA) until the livers began to pale yellow. Then livers were perfused with 5 ml of buffer C (buffer B containing 0.6 mg/ml of CaCl_2_ and 0.4 mg/ml of collagenase type IV) and digested for 20 min at 37°C. Digested livers were shredded and continuously incubated in 5 mL of buffer C at 37°C for another 20 min. Digestion was stopped by adding William’s E medium (Gibco, Grand Island, NY) supplemented with 5% chicken serum and 2 mg/ml of BSA. Cells were collected by filtering the digest sequentially through 200, 75 and 30 µm filters. Cells were incubated with red blood cell lysis buffer for 15 min on ice and then washed with William’s E medium containing 100 U/ml of penicillin-streptomycin and 2 mg/ml of BSA to remove cell fragments and erythrocytes. Cell number and viability were verified by the trypan blue exclusion test. Cells were cultured at a density of 6×10^5^ cells/ml in 12-well plates in William’s E medium supplemented with 5% chicken serum, 100 U/ml penicillin-streptomycin, 10 µg/ml insulin and 30 mM NaCl at 37°C with 5% CO_2_ in a humidified incubator.

### Transfection of Chicken Hepatocytes

Primary chicken hepatocytes were cultured in 12-well plates for approximately 24 h before transfection. Chicken hepatocytes were transfected with 80 nM miRCURY LNA-anti-miR-33 or LNA scramble control (Exiqon, Woburn, USA) utilizing X-tremeGENE HP DNA transfection reagent (Roche, Mannheim, Germany). The expression of miR-33 and *FTO* mRNA was detected 48 h post-transfection.

### Culture and Transfection of C2C12 Cells

C2C12 cells were obtained from Cell Resource Center of Shanghai Institutes for Biological Sciences, Chinese Academy of Sciences. They were maintained in Dulbecco’s Modified Eagle’s Medium (DMEM) containing 2 mM L-Glutamine, 1 mM sodium pyruvate, 100 U/ml of penicillin-streptomycin and 10% fetal bovine serum (FBS) (Gibco) at 37°C with 5% CO_2_ in a humidified incubator. To overexpress miR-33, cells were seeded at a density of 1.5×10^5^ cells/ml in 6-well plates for 24 h and transfected with pcDNA3.1-miR-33 using the X-tremeGENE 9 DNA Transfection Reagent (Roche) as described previously [25]. After 48 h, total RNA was isolated and used to quantify the expression level of miR-33.

To determine if miR-33 targets the *FTO* 3′UTR, C2C12 cells were seeded in 24-well plates for 24 h before transfection. pMIR-FTO (Firefly luciferase) or pMIR-mutFTO, pcDNA3.1-miR-33 or pcDNA3.1-NC-miRNA and transfection efficiency control pRL-CMV (Renilla luciferase) were mixed and co-transfected into the cells using X-tremeGENE 9 DNA Transfection Reagent (Roche). Cells were harvested and lysed 48 h after transfection. Luciferase activity was measured using the Dual-Glo Luciferase Assay System (Promega, Madison, WI) on a Modulus single tube luminometer (Turner BioSystems, Sunnyvale, CA). Firefly luciferase activity was normalized to *Renilla* luciferase activity. This transfection experiment was performed in triplicate wells and repeated at least three times.

### Statistical Analysis

All data are presented as mean ± standard error of the mean (SEM). The statistical significance of differences was evaluated with the student’s t-test or one way ANOVA. *P*<0.05 was considered significant.

## Results

### miR-33 is Predicted from Intron 16 of the Chicken *SREBF2* Gene

**Figure 1:**
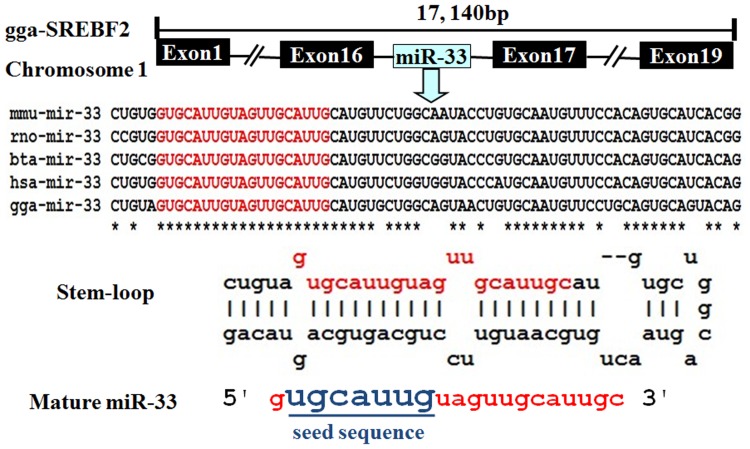
Prediction of transcription of chicken miR-33 from the chicken *SREBF2* gene. A miR-33 stem-loop is predicted from intron 16 of *SREBF2*, and the sequence of this part of the *SREBF2* gene is highly conserved across mammalian species (mmu: mouse; rno: rat; bta: cow; hsa: human) and chicken (gga: chicken).

The miR-33 family has been predicted to be present in several mammalian species, including human, rat, mouse, and cow. In some species there is a single member of this family which gives the mature product miR-33. However, primates and a limited number of other species have two members of this family called miR-33a and miR-33b, which are located in the intronic regions of the *SREBF2* and *SREBF1* genes, respectively. Aligning the chicken *SREBF2* and *SREBF1* DNA sequences with the corresponding human, mouse, rat, and cow sequences revealed that intron 16 of the chicken *SREBF2* gene might encode the chicken miR-33 (Fig. 1). A typical stem-loop pre-miRNA and mature miRNA can be predicted from this region of the chicken genome (Fig. 1).

### Expression of miR-33 and *SREBF2* Gene in Various Chicken Tissues

**Figure 2:**
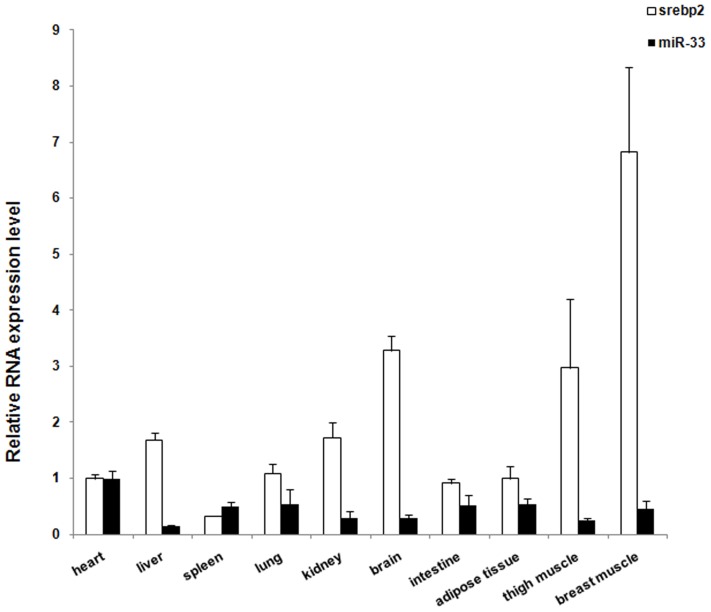
Expression profile of miR-33 and *SREBF2* mRNA in chicken tissues. The expression levels of miR-33 and *SREBF2* mRNA in 10 tissues from 4-wk-old chickens were analyzed by real-time qRT-PCR. The expression of miR-33 was normalized to 18S rRNA, and the expression of *SREBF2* mRNA was normalized to β-actin mRNA. Data are means ± SEM (n = 3 chickens).

The expression of miR-33 in 10 types of tissues from 4 week-old chickens was analyzed using real-time qRT-PCR. miR-33 expression was detected in all 10 chicken tissues with the highest level in the heart (Fig. 2). We also analyzed the expression of the host gene *SREBF2* in the same set of chicken tissues. *SREBF2* mRNA was also widely expressed in chickens, with the highest level in breast muscle (Fig. 2). The expression levels of miR-33 and *SREBF2* mRNA did not parallel in most of the tissues analyzed (Fig. 2). The correlation coefficient (R) between miR-33 and *SREBF2* mRNA expression in different chicken different tissues was −0.268 (*P*>0.05). This suggests that their expressions are not co-regulated in most chicken tissues.

### Computational Prediction of miR-33 Target Genes

To predict the target genes of chicken miR-33, the chicken 3′UTRs were analyzed for potential binding sites of miR-33 by the computational algorithm “miRanda”. Of the 11,891 chicken 3′UTRs in the 3′UTR database, 378 were predicted to be targeted by miR-33. In addition, a variety of online target prediction software was used to predict the targets of miR-33. Top targets of miR-33 (total context score <−0. 30 by TargetScan) are listed in Table 2.

**Table 2:** Computational prediction of partial miR-33 target genes by Targetscan.

Human orthologof target gene	Representativetranscript	Gene name	Conserved sites			Total context+score
			8mer	7mer+m8	7mer+1A	
ABCA1	NM_005502	ATP-binding cassette, sub-family A (ABC1), member 1	*			−0.90
CROT	NM_001143935	carnitine O-octanoyltransferase	*			−0.75
NAA30	NM_001011713	N(alpha)-acetyltransferase 30, NatC catalytic subunit	*			−0.59
GRB10	NM_001001549	growth factor receptor-bound protein 10	*			−0.54
ZNF281	NM_012482	zinc finger protein 281	*			−0.49
NPC1	NM_000271	Niemann-Pick disease, type C1	*			−0.47
VCAN	NM_001126336	versican		*		−0.47
ADCYAP1	NM_001099733	adenylate cyclase activating polypeptide 1 (pituitary)	*			−0.46
GLRA1	NM_000171	glycine receptor, alpha 1		*		−0.44
SLC12A5	NM_001134771	solute carrier family 12, member 5	*			−0.44
IGF1	NM_000618	insulin-like growth factor 1 (somatomedin C)	*			−0.42
SCN8A	NM_001177984	sodium channel, voltage gated, type VIII, alpha subunit	*			−0.41
MRPS25	NM_022497	mitochondrial ribosomal protein S25	*			−0.41
PIM3	NM_001001852	pim-3 oncogene	*			−0.41
CPT1A	NM_001876	carnitine palmitoyltransferase 1A (liver)	*			−0.40
PRKCE	NM_005400	protein kinase C, epsilon	*			−0.40
ICK	NM_014920	intestinal cell (MAK-like) kinase	*			−0.39
ABHD2	NM_007011	abhydrolase domain containing 2	*			−0.38
FGF7	NM_002009	fibroblast growth factor 7	*			−0.37
RAP2A	NM_021033	RAP2A, member of RAS oncogene family	*			−0.37
RMND5A	NM_022780	required for meiotic nuclear division 5 homolog A		*		−0.37
HIPK2	NM_001113239	homeodomain interacting protein kinase 2	*			−0.35
AKAP2	NM_001004065	A kinase (PRKA) anchor protein 2	*			−0.35
PALM2-AKAP2	NM_007203	PALM2-AKAP2 readthrough	*			−0.35
GAS1	NM_002048	growth arrest-specific 1	*			−0.35
PCDH18	NM_019035	protocadherin 18	*			−0.35
TPM3	NM_001043351	tropomyosin 3	*			−0.34
DDX3X	NM_001193416	DEAD (Asp-Glu-Ala-Asp) box polypeptide 3, X-linked			*	−0.34
ZMIZ1	NM_020338	zinc finger, MIZ-type containing 1	*			−0.34
UBE2V2	NM_003350	ubiquitin-conjugating enzyme E2 variant 2	*			−0.33
NAP1L4	NM_005969	nucleosome assembly protein 1-like 4			*	−0.33
SIK1	NM_173354	salt-inducible kinase 1	*			−0.31
KIAA1409	NM_020818	KIAA1409	*			−0.30
GRIA3	NM_000828	glutamate receptor, ionotrophic, AMPA 3	*			−0.30

**Note:** Target genes are listed in the table of that whose total context score is lower than −0.30. Interacting sites with miR-33 in the 3′UTR of predicted target genes are in parentheses. 8 m: An exact match to positions 1–8 of miR-33; 7m+m8: An exact match to positions 2–8 of miR-33; 7m+1A: An exact match to positions 2–7 of miR-33 followed by an ‘A’.

### Verification of the Interaction between miR-33 and the *FTO* 3′UTR

One of the predicted miR-33 targets is the *FTO* gene. We chose to experimentally validate the physical and functional interaction between miR-33 and FTO because the latter was recently discovered to be associated with obesity [15], [22], [23], [26], and because this interaction has not been characterized in any species.

**Figure 3:**
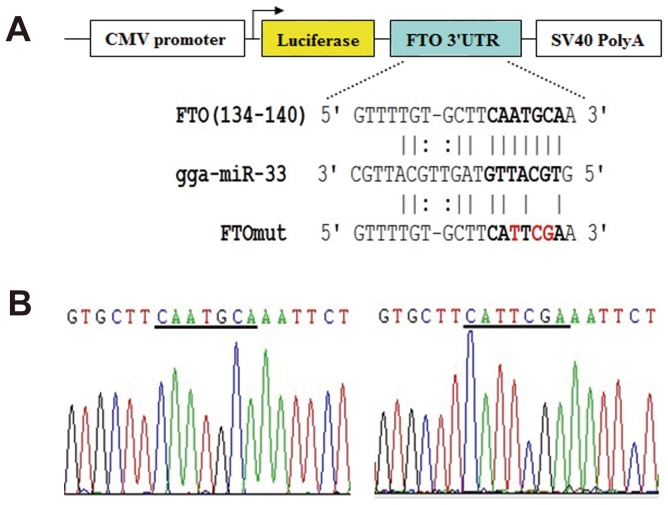
Construction of the pMIR-FTO and pMIR-FTOmut plasmids. The top panel shows the structure and cloning sites of the pMIR-reporter vector. Wild type and the miR-33 binding site-mutated *FTO* 3′UTR were cloned into the reporter vector. The middle panel shows complementarity between miR-33 and predicted target site in the *FTO* 3′UTR. The bottom panel shows the sequences of either wild type or mutant *FTO* 3′UTR.

**Figure 4:**
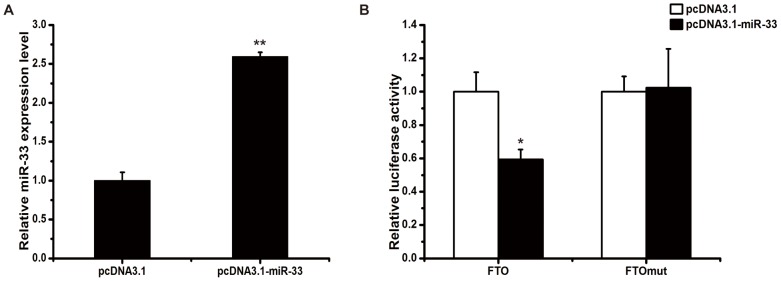
Verification of the interaction between miR-33 and the *FTO* 3′UTR. A: Verification of over-expression of miR-33 in C2C12 cells. C2C12 cells were transfected with the control vector pcDNA3.1 or the miR-33 over-expression vector pcDNA3.1-miR-33. The expression level of miR-33 was detected by real-time qRT-PCR. Data are expressed as means ± SEM (n = 3). ** *P*<0.01. B: Reporter gene analysis of the interaction between miR-33 and *FTO* 3′UTR. C2C12 cells were co-transfected with pMIR-FTO or pMIR-FTOmut and pcDNA3.1 or pcDNA3.1-miR-33. Data are expressed as means ± SEM (n = 3). * *P*<0.05.

To determine whether the putative miR-33 target sequence in the *FTO* 3′UTR mediates translational repression by miR-33, we inserted the 3′UTR of the chicken *FTO* transcript downstream of a luciferase reporter gene to generate the reporter plasmid pMIR-FTO (Fig. 3). We also constructed a similar plasmid, pMIR-FTOmut, in which the putative miR-33 binding site in the *FTO* 3′UTR was partially mutated, and a chicken miR-33 over-expression vector named pcDNA3.1-miR-33. We transfected C2C12 cells with the pMIR-FTO or pMIR-FTOmut reporter vector, and pcDNA3.1-miR-33 or pcDNA3.1 (empty vector). Successful overexpression of miR-33 was validated by real-time qRT-PCR (Fig. 4A). Co-transfection of pcDNA3.1-miR-33 resulted in a decrease in luciferase activity expressed from pMIR-FTO, compared with co-transfection of pcDNA3.1 (*P*<0.05, Fig. 4B). This decrease was abolished by mutation of the miR-33 binding site in the *FTO* 3′UTR (Fig. 4B). These results indicate that miR-33 can inhibit FTO expression by directly interacting with the predicted target site in the *FTO* 3′UTR.

### miR-33 Knockdown Up-regulated *FTO* mRNA Expression in Primary Chicken Hepatocytes

**Figure 5:**
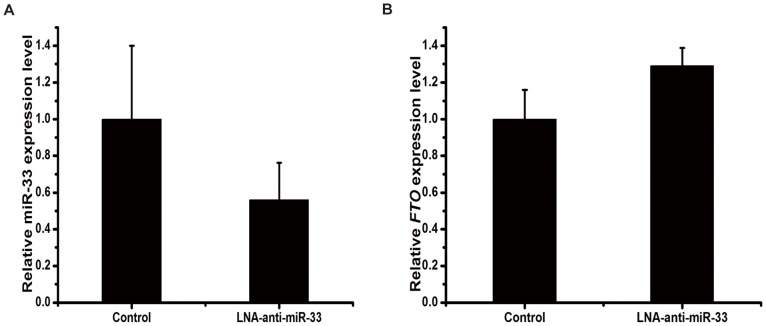
Effect of miR-33 knockdown on the expression of miR-33 and *FTO* mRNA in primary chicken hepatocytes. A: Expression levels of miR-33. Primary chicken hepatocytes were transfected with LNA-anti-miR-33 or LNA scramble control. miR-33 and *FTO* mRNA were quantified by real-time qRT-PCR 48 h after transfection. Data are means ± SEM (n = 3), P = 0.4. B: Expression levels of *FTO* mRNA. Data are means ± SEM (n = 3), P = 0.2.

The *FTO* gene appears to play a role in lipid metabolism and energy homeostasis [23], [27]. *De novo* fatty acid synthesis in chickens takes place mainly in the liver [28]. Thus, in chickens, the liver might be the tissue where the *FTO* gene is involved in lipid metabolism and energy homeostasis. In view of this possibility, we evaluated the interaction between miR-33 and *FTO* mRNA in primary chicken hepatocytes. Specifically, we determined if knockdown of miR-33 expression by LNA-anti-miR-33 would increase *FTO* mRNA expression in primary chicken hepatocytes. Transfection of LNA-anti-miR-33 into chicken hepatocytes decreased miR-33 expression by 44% (Fig. 5A). This decrease was associated with a 29% increase in *FTO* mRNA expression (Fig. 5B). These data suggest the possibility that miR-33 negatively regulates the expression of *FTO* mRNA in chicken liver.

### Inverse Correlation of miR-33 and *FTO* mRNA Expression in Chicken Liver at Different Developmental Stages

**Figure 6:**
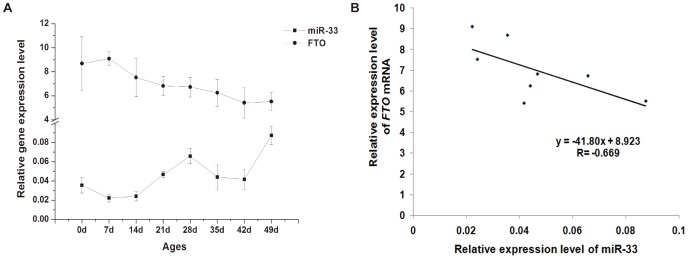
Expression levels of chicken miR-33 and *FTO* mRNA in chicken liver at different postnatal ages. A. The expression levels of miR-33 and *FTO* mRNA in chicken liver from 0 to 49 d of ages were analyzed by qRT-PCR. The former was normalized to 18S rRNA, while the latter to β-actin mRNA. Data are means ± SEM (n = 3). B. Expression levels of chicken miR-33 and *FTO* mRNA in liver from 0 to 49 d of ages are negatively correlated (P = 0.07), as determined by a regression analysis.

To further evaluate the possibility that FTO expression is negatively regulated by miR-33 in chicken liver, we quantified miR-33 and *FTO* mRNA in chicken liver at 8 different ages using real-time qRT-PCR. We found that the expression of miR-33 was increased, whereas that of *FTO* mRNA was decreased from 0 to 49 days of age (Fig. 6A). The correlation coefficient between miR-33 and *FTO* mRNA expression in chicken liver at different developmental stages was −0.669 (*P* = 0.07)(Fig. 6B). These inverse changes in miR-33 and FTO mRNA expression suggest that miR-33 may be one of the negative regulators of *FTO* mRNA expression in the chicken liver during development.

## Discussion

The majority of the characterized miRNA genes are intergenic or oriented antisense to neighboring genes and are therefore suspected to be transcribed as independent units [29]. However, some mammalian miRNAs are located within introns of protein-coding genes or even in exons of long nonprotein-coding transcripts rather than in their own unique transcription units [30]. Intronic miRNAs are typically coordinately expressed and processed with the precursor mRNA in which they reside [31]. miR-33 is an intronic miRNA, and its expression levels paralleled those of its host gene *SREBF2* in diverse cell types, including hepatocytes and macrophages in the human and mouse [8], [10]. In the present study we predicted computationally and validated experimentally the transcription of miR-33 from intron 16 of the chicken *SREBF2* gene. However, our expression data did not support co-regulation of *SREBF2* and miR-33 expression across 10 types of chicken tissues examined.

Predicting targets is an important first step to determine the function of a miRNA. Many algorithms and databases for miRNA target predictions have been established, and among them, miRanda [25], TargetScan [25], [32], and PicTar [33], appear to be the most widely used miRNA target prediction methods. In this study, 378 genes were predicted as the target genes of miR-33 among the total 11,891 chicken genes within the 3′UTR database using “miRanda”. The “TargetScan” principle was also applied in the prediction procedures: the target site should match to the seed region of miRNA (nucleotides 2–7), the 8^th^ nucleotide of miRNA should also be a match or the target nucleotide corresponding to the first nucleotide of miRNA should be an A [32]. One of the predicted target genes of miR-33 named *FTO* is a member of the non-heme dioxygenase superfamily, and has been recently implicated in regulation of lipid and energy metabolism [22], [23]. Dual-luciferase reporter assays and site mutation analyses validated that chicken *FTO* was a target gene of miR-33. Because in chickens *de novo* fatty acid synthesis occurs primarily in the liver, we further studied the possibility that miR-33 targets *FTO* in the chicken liver. One of the most powerful and straightforward ways to determine the relationship between a miRNA and a mRNA in tissues or cells is to determine the effect of knockdown of the miRNA on the expression of the mRNA of interest. Using LNA-anti-miR-33, we successfully reduced the expression of endogenous miR-33 in primary chicken hepatocytes, and this reduction was associated with an up-regulated expression of *FTO* mRNA. This association supports that the *FTO* gene is targeted by miR-33 in chicken hepatocytes. We also observed that miR-33 and *FTO* mRNA expression were inversely correlated in chicken liver at most of the developmental ages examined. This inverse relationship further supports the possibility that miR-33 negatively regulates FTO expression in chicken liver. At day 35 and day 42 of age, the expressions of miR-33 and *FTO* mRNA were not inversely correlated. This suggests that the expression of *FTO* at these two stages may be regulated predominantly by mechanisms other than miR-33.

In the chicken, *FTO* is widely expressed. Expression of *FTO* in the hypothalamic nuclei involved in energy balance regulation has been shown to respond to nutritional manipulations such as feeding and fasting [34]–[36]. Fasting has been shown to also increase *FTO* gene expression in the cerebrum, liver, breast muscle and subcutaneous fat. Alterations in feeding status resulted in significant changes in *FTO* expression in the liver, but not in other tissues of broiler chickens [37]. In addition to this, hepatic *FTO* expression changes in response to metabolic states, and glucose reduces hepatic *FTO* mRNA expression independently of body weight [27]. Since miR-33 inhibits the expression of *FTO*, it might play a role in mediating the nutritional regulation of *FTO* expression in chicken liver.

In conclusion, chicken miR-33 is transcribed from intron 16 of the chicken *SREBF2* gene and is expressed in various chicken tissues. miR-33 might be involved in lipid metabolism and energy homeostasis in the chicken by negatively regulating the expression of the *FTO* gene in the liver.
